# Visualization tools for human structural variations identified by whole-genome sequencing

**DOI:** 10.1038/s10038-019-0687-0

**Published:** 2019-10-30

**Authors:** Toshiyuki T. Yokoyama, Masahiro Kasahara

**Affiliations:** Department of Computational Biology and Medical Sciences, Graduate School of Frontier Sciences, The University of Tokyo, Chiba, Japan

**Keywords:** Genome informatics, Genetic databases

## Abstract

Visualizing structural variations (SVs) is a critical step for finding associations between SVs and human traits or diseases. Given that there are many sequencing platforms used for SV identification and given that how best to visualize SVs together with other data, such as read alignments and annotations, depends on research goals, there are dozens of SV visualization tools designed for different research goals and sequencing platforms. Here, we provide a comprehensive survey of over 30 SV visualization tools to help users choose which tools to use. This review targets users who wish to visualize a set of SVs identified from the massively parallel sequencing reads of an individual human genome. We first categorize the ways in which SV visualization tools display SVs into ten major categories, which we denote as view modules. View modules allow readers to understand the features of each SV visualization tool quickly. Next, we introduce the features of individual SV visualization tools from several aspects, including whether SV views are integrated with annotations, whether long-read alignment is displayed, whether underlying data structures are graph-based, the type of SVs shown, whether auditing is possible, whether bird’s eye view is available, sequencing platforms, and the number of samples. We hope that this review will serve as a guide for readers on the currently available SV visualization tools and lead to the development of new SV visualization tools in the near future.

## Introduction

Structural variations (SVs) are defined as large variations, which are often 50 bp or longer [1, 2]. SVs are known to be associated with human traits, genetic diseases, or cancers [3, 4], and therefore identifying SVs plays an important role in genome analysis. To identify SVs in a whole genome, the following steps are usually performed: (1) library preparation and whole-genome shotgun sequencing, (2) aligning the shotgun reads, (3) SV identification (SV call), (4) SV annotation, and (5) SV visualization [5]. The last step, SV visualization, is a critical step in the SV analysis; below, we explain the importance of SV visualization tools in the entire SV analysis.

Massively parallel sequencing technologies have enabled the de novo detection of SVs of varying sizes [6]. Although SV identification using the second-generation sequencing (often referred to as the next-generation sequencing) technologies has suffered from an overwhelmingly large amount of false positives due to their short read length, comprehensive de novo SV detection are realistic [7, 8], which was impossible with the older sequencing technologies. With the advent of third-generation sequencing technologies that provide us with long reads, more complex SVs are expected to be identified because longer reads are more easily aligned to the reference genome. Indeed, recent studies using long-read sequencing even revealed 3-hop fusion genes and the ﻿long-range structure of chromothripsis [9, 10]. Further, whole-genome sequencing using the third-generation sequencing technology identified around 20,000 SVs against the reference genome per human genome [8, 11–13]. Because the outputs of SV callers still include many false positives/negatives [14, 15], the manual inspection of tens of thousands of SVs using read alignments and genomic annotations is often needed to filter out false positive SVs. The visualization of SVs is a critical step for interpreting their potential impacts.

To visualize SVs, one has to consider several different aspects, depending on the research goals; for example, the choice of tools depends on which sequencing platforms the data includes. However, there are more than 30 SV visualization tools, and users often have difficulties choosing the right tool. The way the SVs are visualized varies from tool to tool; they provide fundamentally different views based on different design concepts and on different research goals.

Here, we provide the first comprehensive review specifically for SV visualization tools in the era of long-read sequencing, such as PacBio Sequel II or Oxford Nanopore Technologies MinION/PromethION. There are already several technical reviews for the SV-calling process [5, 16], which includes a survey of SV visualization tools, but which does not reflect the recent development of dozens of SV visualization tools. We focused on SV visualization tools for visualizing a set of SVs identified from massively parallel sequencing reads of one or more human genomes but not limited to, regardless of the sequencing platforms (short reads, long reads, or optical mapping), although proprietary tools and unpublished tools might have been missed.

We note that we omitted the following types of tools: (1) Visualization tools for metagenomes, pangenomes, or alternative splicing revealed by RNA-sequencing; the currently available implementations are not designed for SVs. (2) Visualization tools for assembly graphs are not designed for large genomes (e.g., human genomes); some of them are too slow, and others can display a whole genome at once, so the displayed graphs would be too complex and uninterpretable (so-called “hairball problem”); the rest of those tools are not able to visualize genomic annotations and/or read alignments, which is a non-negligible restriction for visualizing SVs. (3) Synteny browsers for comparing multiple genomes across species; they do not focus on independent SVs in human genomes.

We hope that this paper will help readers choose the right SV visualization tools for their research goals, so that they may be able to focus on biological questions rather than spending their time surveying dozens of SV visualization tools.

## SV visualization

### View modules for visualizing SV

**Fig. 1:**
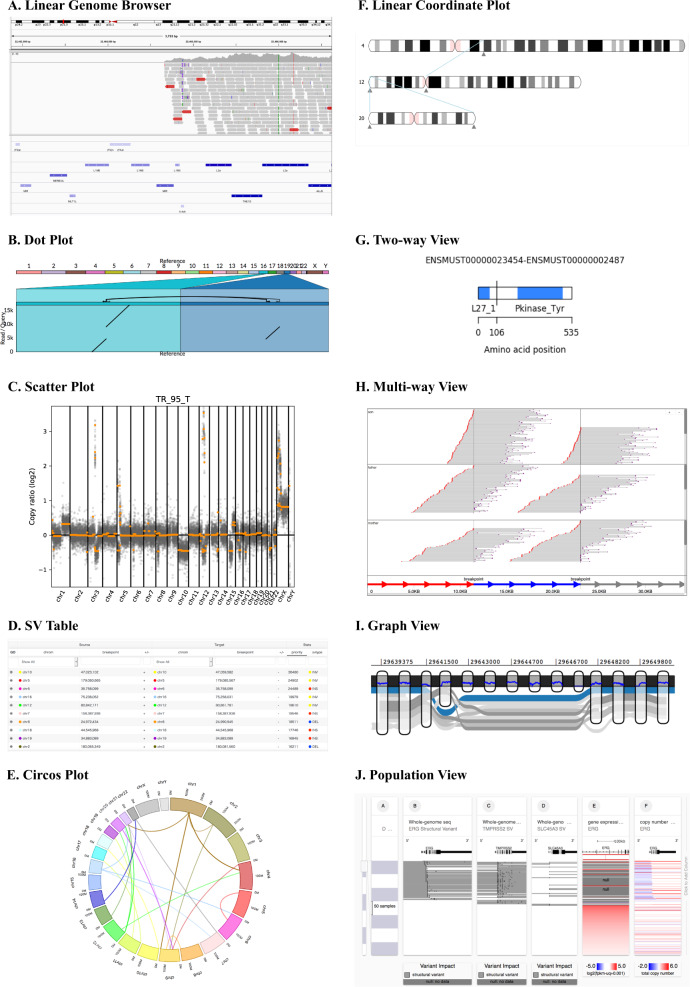
Screenshots of view modules. **a** Linear Genome Browser (here, IGV) shows Illumina read alignments and annotations for a cancer cell line, LC-2/ad: a large deletion in chromosome 9 with read alignments (in the middle) and repeat annotations (in the bottom) is shown. Alignments courtesy of Mr. Sarun Sereewattanawoot and Prof. Yutaka Suzuki. **b** Dot plot implemented in Ribbon shows the split-read alignment between a long PacBio read and the two regions on the human genome: a breast cancer cell line, SK-BR-3, exhibits a translocation between chromosome 16 and 19. **c**. Scatter plot in CNVKit shows the distribution of copy number alterations identified by Illumina reads: a semi-log scatter plot shows the normal-tumor copy ratio across all chromosomes in a melanoma sample. **d** SV table in MoMI-G for filtering and navigation: a list of SVs identified in CHM1, a hydatidiform mole genome. **e** Circos plot shows inter-chromosomal SVs identified by using 10x Chromium: SVs between the human reference genome and the NA12878 sample. **f** Linear Coordinate plot shows SVs between the NA12878 sample and the human reference genome: SVs between chromosome 4, 12, and 20 are shown. **g** Two-way view in AGFusion shows two fusion partner genes with details on each side: a fusion gene across DLG1 and BRAF (535 amino acids in total) in the mouse genome. **h** Multi-way view in svviz shows Illumina read alignments along three genomic intervals: the two breakpoints are suggested by the paired-end mapping of the Illumina reads from a trio sample (HG002/003/004). **i** Graph view in MoMI-G (implemented by using SequenceTubeMap) shows a graphical view of a genomic region with long-read alignments: a deletion of >200 bp is identified by the read alignments. **j** Population view in UCSC Xena shows SV calls, gene expression, and the copy numbers of multiple genomes: copy numbers on ERG across more than 300 samples represented as a heatmap

There are many visualization methods for SVs and many have significant overlaps in the ways they display data. We organized the displaying methods into ten major categories (Table 1; Fig. 1), namely, linear genome browser, dot plot, scatter plot, SV table, linear coordinate plot, circos, two-way view, multiway view, graph view, and population view. We will denote them as view modules throughout this paper.

**Table 1:** The characteristics and targets of various view modules

Category	Whole genome (inter-chromosome)	Chromosome	Gene	Nucleotide	Purpose	Examples
Linear Genome Browser	–	Yes	Yes	Yes	Read alignment, read coverage, and/or gene annotations	IGV [20, 21], JBrowse [18, 19]
Dot plot	Yes	Yes	Yes	Yes^a^	Alignment between a pair of sequences	Assemblytics [25], Loupe
Scatter plot	Yes	Yes	Yes	Yes	Copy number against the reference genome	iCopyDav [28], CNVKit [27]
SV table	Yes	Yes	–	–	Navigation through SVs	SplitThreader [35], MoMI-G [37]
Circos plot	Yes	Yes	–	–	Bird’s eye view of SVs	Circos [29], CIRCUS [49]
Linear coordinate plot	Yes	Yes	–	–	Bird’s eye view of SVs	Fastbreak [30], Gremlin [51], ViVar [52]
Two-way view	–	–	Yes	Yes	Read alignment/coverage and gene annotations for fusion genes	Loupe, iFUSE [53], targetSeqView [47], AGFusion [58], BreakPoint Surveyor [59], and MAVIS [57]
Multi-way View	–	–	Yes	Yes	Read alignment/coverage and gene annotations for (possibly complex) fusion genes	svviz [36], Ribbon [26]
Graph view	–	–	Yes	Yes	Structure of SV, read alignment/coverage, and gene annotations	MoMI-G [37], GfaViz [38]
Population view	a	Yes	Yes	Yes	Copy number or SV frequencies over population	SV-Pop [54], UCSC Xena [55]

^a^Yes, in theory, but no available tools in practice

Linear genome browsers, such as UCSC genome browser [17], JBrowse [18, 19], and Integrative Genomics Viewer (IGV) [20, 21] (Fig. 1A), display a genomic interval of a reference genome and nucleotide sequence horizontally, and various types of custom tracks superimposed over the reference genome as parallel lines. Custom tracks are displayed in various ways. For example, read alignments are displayed as pileups of the reads against the aligned genomic interval, gene/repeat annotations are displayed as rectangles to represent the range on reference genomes, and read coverages are displayed as a line or bar chart, whose x-axis is the reference genome. Since the linear genome browsers were designed when long-read sequencing, now critical for SV identification, was not available, linear genome browsers are not the most suitable for visualizing SVs, especially when the SVs are not small deletions. However, linear genome browsers provide a sophisticated visualization for read alignments that is not usually available in other types of view modules, such that linear genome browsers are useful when we need individual alignments. The recent updates of IGV include supports for viewing large-scale SVs, such as linking split alignments and showing insertion sequences, among others [22]. Several SV visualization tools for the manual reviewing of SVs use IGV as a backend tool for generating the screenshots of the alignments around the SVs. Web-based genome browsers (or libraries), such as JBrowse [18], igv.js (https://github.com/igvteam/igv.js/), Dalliance [23], or pileup.js [24], provide view modules that are embeddable in future SV visualization tools; users can create a custom web-based tool with the full power of linear genome browsers to display individual read alignments and annotations.

Dot plot (Fig. 1B) has been commonly used in the field of comparative genomics for drawing alignments between two given sequences. Recent tools such as Assemblytics [25] or Ribbon [26] support dot plots to show the nucleotide-level alignments to whole genome alignments for displaying SVs of arbitrary sizes. In a dot plot, the X- and Y-axis represent two sequences to be compared. Alignments between the two sequences are shown as diagonal segments, each of which represents a single alignment that starts/ends at the positions projected on the *X*- and *Y*-axis.

Scatter plot (Fig. 1C) is often used to describe the difference in the copy number of genomic segments over chromosomes. Here, we define a scatter plot for copy number variations (CNVs) as a figure in which the estimated copy numbers for genomic segments are shown as the Y-coordinate of points, lines, or bars along genomic segments on the *X*-axis. Scatter plot is implemented in several CNV visualization tools such as CNVKit [27] or iCopyDAV [28]. This view module is useful for quickly capturing the genome-wide distribution of CNVs.

A simple table is also commonly used for showing a list of SVs. Here, we denote this as SV table (Fig. 1D). SV Table is simple but provides a critical navigation method for users. For example, when users wish to inspect all SV candidates output by an SV identification tool, clicking on the SV table would quickly take users to individual SVs without asking users to manually input the chromosome numbers or positions. SV table often is equipped with a filtering function.

Circos plot (Fig. 1E) has many applications for visualizing genome features [29]. In the context of the SV visualization of human genomes, chromosome 1–22, X, and Y are arranged as arcs of a circle, and the SVs are represented by the curves. This view module is useful for viewing large SVs when the number of the large SVs is small enough so that we can recognize them.

Linear coordinates plot (Fig. 1F) displays one or more chromosome (often karyotypes) and SVs. Chromosomes are vertically or horizontally placed, and lines or curves connect the two-end points of SVs. Linear coordinates plot that displays multiple chromosomes in parallel is often known as a parallel coordinates plot [30] in the context of SV visualization. This view module is also useful for viewing large intra-chromosomal SVs or inter-chromosomal SVs.

Two-way view (Fig. 1G) is a concept that is applied to other view modules, such as linear genome browser. Two-way view is used, for example, for displaying fusion genes, for which we need to display two distant genomic intervals involved in the fusion. The essential feature of two-way view is that two (but no more) genomic intervals are shown in a single panel. The two intervals are usually arranged side by side or may occasionally be the *X*- and *Y*-axis (c.f., in dot plot). Gene annotation, read coverages, read alignments, and isoforms on the two intervals may be shown along the two intervals.

Multiway view (Fig. 1H) is an extended concept of two-way view; multiway view enables us to visualize more than two intervals of read alignments/coverage on a single panel. The intervals are arranged along an axis and read alignments or coverages may be shown along the intervals. Multi-way view often assumes long reads that may align with multiple distant locations on the genome, whereas two-way view often assumes short reads that may span up to two locations.

Graph view (Fig. 1I) is a view based on graph genome. Graph genome is an emerging approach for representing SVs by embedding them in a mathematical graph that models genomes and SVs as nodes and edges. There are three reasons to use graph genomes and graph view. First, variant calling using linear reference genomes is known to have a bias toward the reference alleles [10, 11], but variant calling using graph reference genomes ﻿effectively eliminates this bias [31]. Second, displaying heterozygous and large SVs in existing (linear) genome browsers is difficult because the implementation of existing linear genome browsers implicitly assumes that a target genome has only small variants to the reference genome. Third, to our knowledge, there is no linear genome browser that displays nested SVs in a way that they can be easily recognized as nested. Graph genome and graph view provide a natural way to understand the structure of SVs, regardless of their size, their originating chromosomes, SV types (insertion/deletions/etc.), or the existence of nesting SVs. Because the development of graph genome algorithms is still in the early stage, the way in which graph view modules display genomes and SVs is expected to change quickly over time. Therefore, users who prefer stable and robust implementations may not wish to use graph view. Nevertheless, graph view is expected to deliver more accurate, natural, and unbiased ways for users to understand the complex natures of SVs in human genetics, population genetics, and cancer genetics.

*Population view* (Fig. 1J) displays multiple tracks, each of which represents either an individual genome or a representative genome of a group. It is specifically designed to visualize population genomes, rather than focusing on a single human genome. A possible implementation could be a heatmap, where each row represents a single individual/group and each column represents a single variant.

### SV visualization tools

We define SV visualization tools as tools for visualizing one or more SVs. Because a variety of SV visualization tools have been developed by researchers worldwide to achieve slightly different research goals, the best choice of SV visualization tool for the end users depends on many factors.

To help readers choose which SV visualization tools to use, in the following, we review SV visualization tools (Table 2; Supplementary Table 1) based on various criteria, including but not limited to (1) what sequencing platforms are used, (2) if graph genomes are the preferred way for representing genomic variants, (3) if CNVs are to be examined, (4) if manual reviewing of SVs is needed, (5) if the bird’s eye view of SVs is needed, and (6) if the number of samples is in the hundreds or thousands.

**Table 2:** The curated list of SV visualization tools

Program	Publication year	Type	Programming language	License	Recent activities in GitHub	BAM input	Explicit long-read support	VCF input	Installation	Linear genome browser	Dot Plot	Scatter plot	SV table	Circos plot	Linear coordinate plot	Two-way view	Multi-way view	Graph view	Population view	Sequencing platform
Integrative genomics viewer	2011	Commandline/Standalone	Java	MIT	✓	✓	✓	✓	Binary	✓	✗	✗	✗	✗	✗	✗	✓	✗	✗	Illumina, long reads
New genome browser	2017	Web app	Java, JavaScript	MIT	✓	✓	✗	✓	Docker image or binary	✓	✗	✗	✓	✗	✓	✗	✓	✗	✗	Illumina
BasePlayer	2018	Standalone	Java	AGPL-3.0	✓	✓	✓	✓	Installer or binary	✓	✗	✗	✓	✗	✗	✗	✓	✗	✓	Illumina, long reads
Ribbon	2016	Web app	JavaScript	MIT	✗	✓	✓	✗	Git clone	✓	✓	✗	✓	✗	✗	✗	✓	✗	✗	Long reads
SplitThreader	2016	Web app	JavaScript	MIT	✗	✗	✗	✓	Git clone	✗	✗	✗	✓	✓	✓	✗	✗	✗	✗	^f^
svviz	2015	Commandline/Web app	Python	MIT	✓^e^	✓	✓	✓	pip (Python package)	✓	✗	✗	✗	✗	✗	✗	✓	✗	✗	Illumina, long reads
Samplot	N/A	Command-line/Web app	Python	MIT	✓	✓	✓	✓	Git clone	✓	✗	✗	✓	✗	✗	✓	✗	✗	✗	Illumina, long reads
MoMI-G	2019	Web app	TypeScript	MIT	✓	✓	✓	✓	Git clone	✓	✗	✗	✓	✓	✗	✗	✗	✓	✗	Long reads
GfaViz	2018	Commandline/Standalone	C + +	ISC^a^	✓	✗	✗	✗	Git clone	✗	✗	✗	✗	✗	✗	✗	✗	✓	✗	^f^
gGnome	N/A	Library	R	MIT	✓	✗	✗	✓	devtools (R library)	✗	✗	✓	✗	✗	✗	✗	✓	✓	✗	Illumina
FastBreak	2012	Web app	JavaScript, Python	Unknown	✗	✓	✗	✗	Git clone	✗	✗	✗	✓	✓	✓	✗	✗	✓	✗	Illumina
cnvCurator	2015	Standalone	Java	LGPL	N/A	✓	✗	✗	Binary	✓	✗	✗	✓	✗	✗	✗	✓	✗	✗	Illumina
CNView	2016	Commandline	R	MIT	✗	✗	✗	✗	Git clone	✗	✗	✗	✗	✗	✓	✗	✗	✗	✗	Illumina
CNVkit	2016	Commandline	Python	Apache-2.0	✓	✓	✗	✗	Docker image, pip (Python package)	✗	✗	✓	✗	✗	✗	✗	✗	✗	✓	Illumina
iCopyDAV	2018	Commandline	R, Shell	Unknown	✗	✓	✗	✗	Git clone	✗	✗	✓	✗	✗	✗	✗	✗	✗	✗	Illumina
VIPER	2018	Web app	Java, JavaScript	GPL-3.0	✓	✓	✓	✓	Binary	✓	✗	✗	✓	✗	✗	✗	✗	✗	✗	Illumina, long reads
SVCurator	2019	Web app	Python, JavaScript	MIT	✗	✓	✓	✓	Unspecified	✓	✗	✗	✗	✗	✗	✗	✓	✗	✗	Illumina, long reads
SV-plaudit	2018	Web app	Python	MIT	✓	✓	✓	✓	Git clone	✓	✗	✗	✗	✗	✗	✓	✗	✗	✗	Illumina, long reads
SVPV	2017	Commandline/Standalone	Python, R	MIT	✗	✓	✗	✓	Git clone	✗	✗	✗	✗	✗	✗	✓	✗	✗	✗	Illumina
targetSeqView	2014	Library	R	GPL	✗	✓	✗	✗	devtools (R library)	✓	✗	✗	✗	✗	✗	✓	✗	✗	✗	Illumina
Circos	2009	Command-line	Perl	GPL	N/A	✗	✗	✗	CPAN (Perl module), official page	✗	✗	✓	✗	✓	✗	✗	✗	✗	✗	^f^
Seqeyes	2011	Web app	Adobe Flash	–		✗	✗	✗	Unavailable	✗	✗	✗	✗	✓	✗	✗	✗	✗	✗	^f^
CIRCUS	2014	Library	R	–		✓	✗	✗	Unavailable	✗	✗	✓	✗	✓	✗	✗	✗	✗	✗	^f^
paplot	2017	Web app	JavaScript	MIT	✓	✗	✗	✗	Git clone	✗	✗	✓	✗	✓	✗	✗	✗	✗	✗	^f^
ViVar	2014	Web app	PHP	Unknown	N/A	✓	✗	✗	Unavailable	✗	✗	✓	✓	✗	✓	✗	✗	✗	✗	Illumina
Gremlin	2010	Web app	JavaScript	–					Unavailable	✗	✗	✗	✗	✗	✓	✗	✗	✗	✗	^f^
Loupe	N/A	Standalone	–	Other^b^	N/A	✗	✗	✗	Binary	✓	✓	✗	✓	✗	✗	✓	✗	✗	✗	10x Chromium
Bionano Access	N/A	Web app	–	Other^c^	N/A	✗	✗	✓	Binary	✓	✗	✗	✓	✓	✓	✗	✗	✗	✗	Bionano Saphyr
SV-Pop	2019	Standalone	Python, R	MIT	✓	✓	✗	✓	Git clone	✗	✗	✗	✗	✗	✗	✗	✗	✗	✓	^f^
UCSC Xena	2019	Web app	JavaScript	Apache-2.0	✓	✗	✗	✗	Installer	✗	✗	✗	✓	✗	✗	✗	✗	✗	✓	^f^
Assemblytics	2016	Web app	Python, R	Unknown	✓	✗	✗	✗	Git clone	✗	✓	✗	✗	✗	✗	✗	✗	✗	✗	^f^
MAVIS	2018	Command-line	Python	Academic^d^	✓	✓	✗	✓	pip (Python package)	✗	✗	✗	✗	✗	✗	✓	✗	✗	✗	Illumina
AGFusion	2016	Commandline	Python	MIT	✓	✗	✗	✗	pip (Python package)	✗	✗	✗	✗	✗	✗	✓	✗	✗	✗	^f^
iFUSE	2013	Web app	PHP, R	–					Unavailable	✗	✗	✗	✗	✗	✗	✓	✗	✗	✗	^f^
Breakpoint Surveyor	2017	Commandline	R, Python, BASH	GPL-3.0	✗	✓	✗	✗	Git clone	✗	✗	✓	✗	✗	✗	✓	✗	✗	✗	Illumina

Availability is as of 2019 May. Recent activities in GitHub are defined as any updates by owners on the master branch, issues, or pull requests at least once after 2018 May

^a^﻿Functionally equivalent to a two-term BSD copyright

^b^Proprietary license (10x Genomics End User Software License)﻿

^c^Proprietary license (Bionano Access End User License)﻿

^d^BC CANCER AGENCY SOFTWARE LICENSE

^e^Recent updates are seen in svviz2 (https://github.com/nspies/svviz2)

^f^Platform independent; read alignments are not visualized

### Traditional linear genome browser

The starting point for most users is integrative genomics viewer (IGV) [20, 21]. IGV has long been used for the visualization of genomic annotations and read alignments, and therefore IGV has the largest user base among genome browsers. Users can easily display various kinds of annotations, such as gene annotations, repetitive elements, histone marks, gene expressions, and conservation levels, as well as read alignments, with explicit support for long reads. Furthermore, IGV has a built-in list of URLs from which users can download the common annotation data such as the GENCODE genes in GRCh37; users do not have to specify the individual URLs for the common annotation data. Therefore, users may be able to find the biological implications of identified SVs faster than with other tools. The implementation is mature and stable; there are many articles and books describing how to use IGV, and therefore many users are already familiar with IGV. Among other linear genome browsers, New Genome Browser (NGB) [32] and BasePlayer [33] are notable for SV analysis. NGB can help users navigate quickly through the variants in the list (SV table). BasePlayer shows the split-read alignments of long reads, even for reads that are aligned with distant genomic regions (Multi-way view). BasePlayer also supports multiple samples as input (population view). Other traditional linear genome browsers are not generally recommended. Ensembl and UCSC genome browser are difficult to install on a local system, and the official public web servers cannot be used to work with clinical human genomes due to privacy concerns. JBrowse is not recommended because it lacks explicit support for SVs. Artemis [34] is useful when users wish to annotate gene structures with read alignments and output from analysis or prediction tools.

### Visualizing SV identified by long reads

Long reads, such as PacBio reads or Oxford Nanopore reads, are more likely to be aligned uniquely with the reference genome because those reads are often longer than the lengths of interspersed repetitive sequences in the human genome. This allows the identification of large, complex, and nested SVs that were previously impossible to accurately and robustly find using only short reads. For example, Oxford nanopore reads can be longer than 1 Mb, which can span very large SVs. These ultra-long reads spanning complex or nested SVs may be aligned with more than two separate regions of the reference genome. The new class of SVs found with long reads necessitates the visualization of large, complex, and nested SVs.

To this end, new SV visualization tools specifically designed for SVs identified by long reads have been developed. One mathematically simple way for this purpose is to use mathematical graphs as an underlying data structure for representing genomes with SVs. We introduce this approach in the next subsection. Another approach extends the traditional way of displaying genomes and read alignments to long reads. Ribbon and SplitThreader [26, 35] are a pair of web-based tools for visualizing SVs with various view modules, such as circos plot, linear coordinate plot, and multiway view. BasePlayer provides multiway view for displaying distant genomic intervals spanned by a long-read alignment, whereas svviz [36] and SVCurator provide multiway view for displaying long reads over an SV. Samplot (https://github.com/ryanlayer/samplot) provides two-way view of long-read alignments for translocations and large SVs, and also provides a web interface to browse the plots for a VCF file with SVs.

### Graph genome-based SV visualization tools

**Fig. 2:**
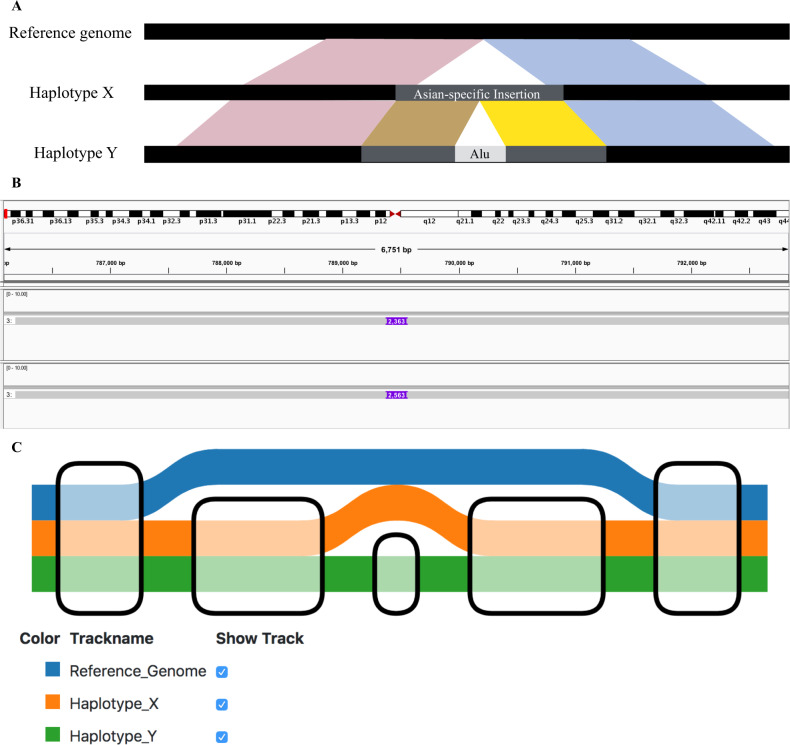
An example of nested insertion. **b** Both haplotype X and haplotype Y have insertion sequences of different sizes against the international reference genome such as GRCh38. Both haplotype X and Y contain the same insertion sequence against the reference genome; for example, imagine that this insertion is an Asian-specific insertion relative to the reference genome. Haplotype Y also contains another insertion, which is often a transposable element. The innermost insertion is a nested insertion relative to the international human reference genome. **b** A typical representation of the nested insertion shown in **a** by linear genome browser. We see the two insertions relative to the international reference genome, but the relationship between the two insertions is not clear. **c** A graph genome representation of the nested insertion shown in **a**. Each node (rounded rectangles) represents a sequence. A colored thick line represents a single haplotype. We can easily see that the two insertions are largely the same except that the insertion on haplotype Y has a small transposable element in it

Visualizing large, complex, and nested SVs using linear genome browsers is difficult. For example, if a genome of Asian origin has a large Asian-specific insertion relative to the international human reference genome, and if one of the haplotypes (haplotype X) contains a small insertion relative to the other haplotype (haplotype Y), the latter haplotype (haplotype Y) has a nested insertion relative to the international human reference genome (Fig. 2). In linear genome browsers, both haplotypes are shown as containing insertions relative to the reference genome. However, the relationship between the two haplotypes cannot be shown, and it is therefore difficult for the user to realize that haplotype A is haplotype B plus a small insertion. Graph genomes can handle large, complex, and nested SVs more naturally than linear genomes, where SVs are represented as the differences from the reference genomes.

MoMI-G [37] provides a graph-based visualization for SVs in human genomes and a customizable layout of view modules with circos plot, SV table, graph view, and linear genome browser. MoMI-G demonstrated that it was able to display nested SVs and a large SV of megabases with long-read alignments in an intuitive way. It may be useful to visualize a smaller region of the human genome, such as the HLA region, GfaViz [38] or graphviz [39], although users have to write a custom script to construct graph genomes to visualize SVs. gGnome (https://github.com/mskilab/gGnome) is an R library that provides graph view for displaying SVs with gene annotations and copy number alterations. Fastbreak [30] is an early work that employed graph view to display multiple distant genomic intervals and their connections (SVs) in a single view.

### Visualizing copy number variations

The best way to visualize SVs in general is still an open question. There are many ways to visualize SVs, but however, to our knowledge, CNVs are always drawn as a scatter plot or a heatmap (Population View). Therefore, visualization tools for CNVs output functionally similar figures.

The visualization tools designed for CNV include cnvCurator [40], CNView [41], CNVKit [27], iCopyDAV [28], and gGnome. The first four tools display copy number alteration on a chromosome by scatter plot. The other tool, gGnome, is able to display intra-chromosomal and inter-chromosomal connections between regions of different copy numbers. Among these tools, cnvCurator is a stand alone tool, provided as a jar file, that supports both tumor-normal analysis and the manual curation of CNVs by looking at the read coverage and alignments. The tools other than cnvCurator are command-line tools for generating publication figures. CNView provides a coverage plot and supports trio analysis. CNVKit supports heatmaps for a hundred genomes (Population View). gGnome visualizes read alignments with copy number and gene annotations on a linear interval.

### Manual reviewing tools for SV

SV identification tools are still in the early stage of their development. Outputs by SV callers often disagree with each other [14, 15]. Therefore, false positive SVs have to be spotted manually by experts. To this end, several groups have developed tools for experts to review SVs for the filtering out of false positive SVs.

VIPER [42] and SVCurator [43] take screenshots of a genome browser for the manual reviewing of SVs; users can easily collect figures without manually launching a genome browser or manually seeking the genomic intervals of SVs. SVCurator employs IGV and svviz2 (the successor of svviz) as its backend, while VIPER employs IGV. Both support long reads and VCF [44] input. SV-plaudit [45] employs samplot as a means to generate figures of SVs, and provides a web interface for rapidly audit SVs, although SV-plaudit requires Amazon S3 and DynamoDB. SVPV [46] shows the range of SV and read coverages against the specific interval of a linear reference genome. targetSeqView [47] provides two-way view to visualize short read alignments.

### Looking at the distribution of SVs

If the user only needs an overview of the SVs in a whole genome, or wishes to quickly view the structure of a given genome without looking at the read alignments, there are several tools available.

Circos [29] has been widely used for visualizing the distribution of SVs. Chromosomes are arranged along a circle, while SVs are drawn as curves that connect the breakpoints of the SVs. Other information, such as gene annotations and read depth, can be displayed with some customization. Because Circos is a general-purpose tool not specifically designed for SVs, and because circos is flexible, the users will need to spend some time writing a configuration file for customizing the output view. There are several circos-based visualization tools, such as Seqeyes [48] and CIRCUS [49], but however, as of writing, they are not currently unavailable. Paplot [50] provides circos plots and a scatter plot that shows the breakpoint distributions of cancer genomes on a web interface.

### Visualization tools for special measurement devices

When measurement devices other than DNA sequencers, such as 10x Chromium or Bionano Saphyr, are used, proprietary browsers specifically designed for the devices are tools that cannot be missed. If a user obtains their sequencing reads using 10x Chromium and has identified SVs, the company’s official browser, Loupe (http://loupe.10xgenomics.com/loupe/), provides multiple view modules, including dot plot, linear genome browser, and two-way view, which are specifically designed for reads barcoded by 10x Chromium. Data obtained using Bionano Saphyr can be displayed with the Bionano’s official browser, Bionano Access (https://bionanogenomics.com/support-page/bionano-access/); it provides circos plot, linear genome browser, and SV table to (implicitly) display SVs. However, these tools do not support reads from other sequencing platforms, so users may have to use other visualization tools, if needed.

### Visualization tools for a few samples

Certain types of analysis, such as trio/pedigree analysis or tumor/normal pair analysis in cancer studies, require genomes to be visualized side by side in order to determine if variants are de novo or not. The following tools allow for the visualization of multiple samples. This subsection is dedicated to tools for the visualization of 2–10 samples; the tools for the visualization of hundreds or even more samples will be discussed in the next subsection.

Modern genome browsers, such as IGV or BasePlayer, can arrange vertically the tracks of both read alignments and identified variants of several genomes, allowing us to compare the several genomes with each other. These full-featured browsers are recommended for general use. There are also tools for visualizing SVs across samples in a more specialized setting. svviz displays the read alignments of multiple samples horizontally. cnvCurator supports tumor/normal analysis. samplot, CNView, SV-plaudit, and SVPV support multiple samples, including trio. Paplot provides circos plots for each sample and a heatmap for mutations across the samples.

### Visualization tools for multiple samples

Since not many research groups visualize SVs in thousands of genomes, the visualization tools for thousands of genomes are not yet mature. For users who wish to analyze hundreds or thousands of genomes, SV-Pop [54], UCSC Xena [55], and BasePlayer are tools that may be of interest. UCSC Xena displays the heatmap of SVs, including copy numbers, across samples with other data types (e.g., gene expression level) in a modern user interface. SV-Pop displays variant frequencies calculated in each population as multiple tracks of line plots. BasePlayer is the only tool in this category that allows users both (1) to easily capture the entire view of thousands of genomes in a heatmap-like genotype matrix and (2) to trace a variant down to read alignments in an individual genome for manual reviewing. BasePlayer may be the only tool for visualizing whole-genome sequencing data of thousands of individuals with underlying raw read alignments. CNVkit can display the copy numbers of dozens of genomes.

### SV analysis by genome comparison

All the tools described so far visualize the SVs identified by comparing the whole-genome shotgun reads and the reference genome. Assemblytics [25] compares two (assembled) genomes without shotgun reads, and displays an interactive dot plot of all-vs-all genome alignments on a web interface.

### visualization tools for fusion genes

Fusion genes that originate from an SV are often searched in cancer genome analysis [56]. MAVIS [57] provides two-way view for visualizing fusion genes with a nifty diagram of exon–intron structures and, optionally, with gene expression levels. AGFusion [58] is a command-line tool for visualizing a fusion gene in two-way view. There are many other visualization tools for fusion genes, but we have omitted them because they are based only on transcripts and do not visualize genomes with SVs.

### Visualization tools in a large analysis pipeline

There is an another class of visualization tools, shipped with large genome analysis pipelines. These visualization tools are specifically designed for those genome analysis pipelines, providing integrated views that show the relationships between SVs and other features, such as read alignment depth, breakpoints, gene annotations, and gene expression levels at the exon level, among others. Breakpoint Surveyor [59] is a set of tools that compares two genomes, such as a virus genome and a human genome, and displays the copy number distributions of two genomes, as well as breakpoint positions and gene expression levels. MAVIS includes a set of tools for clustering, validation, and annotation of SVs, as well as a visualization tool.

## Discussion

In this review, we provided a comprehensive survey of more than 30 SV visualization tools. Because there are many SV visualization tools with significant overlaps in functionality, users can spend hours surveying several tools before they decide which SV visualization tool to use for their project. Here, we first analyzed that how each SV visualization tool displays SVs and related data, before categorizing these into the ten view modules according to the way in which the SV visualization tools display data, including SVs, read alignments, and annotations. We introduced SV visualization tools from a variety of aspects, including the type of SVs, the need for manual auditing, the number of samples, and the type of sequencing platforms. We hope that this paper will serve as a guide for readers when selecting the right tool for their research goals.

The development of SV visualization tools, especially those for long reads or for population genomes, is still in the early phase. Because layout algorithms for rendering graph genomes are not yet mature, rendered graphs are often not easy to interpret by biologists who are not familiar with mathematical graphs. More intuitive views for graph genomes should be developed in the near future. SV analysis methods for population genomes are yet to be explored, and therefore visualization methods for population SVs still need improving.

Although the individual tools we described in this article solve a particular set of visualization issues for SVs, no tool solves all of them simultaneously. For example, gGnome supports displaying phasing and MoMI-G supports displaying read alignments in insertions (i.e., sequences not in the reference genome), but however, no visualization tools support both phasing and read alignments in insertions. We hope that new tools will be developed to solve this problem.

Another point to consider is that we desperately need a standard format for describing SVs. Indeed, the VCF format is designed for this purpose, but there are many ways to describe the same SV in the current VCF specification; as a result, all SV visualization tools can correctly interpret only particular dialects of VCF. For example, the orientation of the breakends of SVs is described either in the ALT field or in the INFO field depending on SV callers, but SV visualization tools usually cannot interpret both ways.

Given the rapid increase in the read length and in the cost reduction of long reads, increasing SVs of various complexities and sizes will be identified by long reads in the near future. Visualization tools for SVs identified by short reads cannot handle sequences that significantly diverge from the reference genomes, which is the reason that they are not suitable for SV analysis based on long reads. Also, the development of SV callers for long reads is needed because existing SV callers do not seem to be able to reveal the entire picture of SVs in the human genome yet and output too many false positives, which hinders the fully automated analysis of SVs in population genomes. The best way to visualize SVs remains an open problem and there is still a great need for new visualization methods.

## Supplementary information

Supplementary Table 1
